# Pharmacokinetic‐pharmacodynamic modelling of risankizumab using chronic plaque psoriasis real‐world data

**DOI:** 10.1002/bcp.70477

**Published:** 2026-02-15

**Authors:** Charlotte M. Thomas, Jessica Ruoheng Wei, David Baudry, Zehra Arkir, Bola Coker, Tejus Dasandi, Kingsley Powell, Monica Arenas‐Hernandez, Jenny Leung, Krystal Rawstron, Chioma Nwaogu, Richard Woolf, Andrew E. Pink, Jonathan Barker, Joseph F. Standing, Catherine H. Smith, Satveer K. Mahil

**Affiliations:** ^1^ King's College London London UK; ^2^ Guy's & St Thomas' Hospital London UK; ^3^ Clinical Biochemistry, Barts Health London UK; ^4^ University College London London UK

**Keywords:** exposure‐response, immune‐mediated inflammatory diseases, pharmacokinetics‐pharmacodynamics, psoriasis, risankizumab

## Abstract

**Aim:**

Risankizumab is a high‐cost biologic treatment for chronic plaque psoriasis, an immune‐mediated inflammatory disease presenting with painful red scaly skin lesions. Inter‐individual heterogeneity in treatment response may be better addressed with personalised rather than fixed dosing. We sought to develop a pharmacokinetic/pharmacodynamic (PK/PD) model to characterise the relationship between risankizumab exposure and treatment response.

**Methods:**

A sequential population PK/PD model was developed using real‐world data (*UK Biomarkers of Systemic Treatment Outcomes in Psoriasis* study) comprising serial PK and Psoriasis Area and Severity Index (PASI) measures. Models were built using R (V4.3.1) and nlmixr2 (V2.1.1.9). One and two‐compartment PK models were tested. A maximal effect turnover model was used to describe PASI, with drug effect on lesion development rate (*K*_*in*_).

**Results:**

The dataset (82 serum risankizumab concentrations; 101 PASI observations) comprised 50 patients with psoriasis (median weight 79.3 kg; age 47 years). PK data were described by a one‐compartment model with first‐order absorption/elimination. Absorption rate (K_*a*_) was fixed from the literature (0.229). Estimated clearance was 0.34 L/day, and volume of distribution 12.9 L. Baseline PASI at model initiation, drug potency (EC_50_) and lesion recovery rate (K_*out*_) were estimated at 23.4, 0.11 mg/L and 0.05 day^*−*1^, respectively.

**Conclusions:**

Pharmacokinetic parameters were similar to risankizumab clinical trials. K_*out*_ estimates aligned with other psoriasis turnover models, highlighting the capture of disease dynamics that may be applied across drugs. This model may inform personalised dosing based on individual patient characteristics, drug exposure and response, to optimise treatment outcomes.

**What is already known about this subject**

- Risankizumab is a high‐cost monoclonal antibody used to treat the common immune‐mediated inflammatory skin disease, chronic plaque psoriasis.
- There are limited exposure‐response models, particularly using real‐world data, exploring the pharmacokinetics/pharmacodynamics (PK/PD) of risankizumab.

**What this study adds**

- We characterise the exposure‐response relationship of risankizumab and Psoriasis Area Severity Index (severity measure), using a turnover model linked with an *E*_*max*_ drug effect model.
- We highlight clinically‐relevant variability between patients in drug potency and skin lesion development rate.
- Our PK/PD model may guide personalised biologic dosing for improved outcomes.

## 1 INTRODUCTION

Chronic plaque psoriasis (herein referred to as ‘psoriasis’) is a lifelong immune‐mediated inflammatory skin condition characterised by red scaly plaques. In Western Europe, psoriasis affects approximately 1–3.5% of the population, with the United Kingdom (UK) exhibiting one of the highest prevalence rates—impacting an estimated 1 million adults.^1^ The introduction of targeted biologic drugs has significantly changed the landscape of psoriasis treatment, enabling exceptional management of the condition. The UK National Institute for Health and Care Excellence (NICE) recommends a total of 12 biologics for the treatment of psoriasis.^2^ These immunomodulators target key inflammatory drivers within the immune response, including interleukin (IL)‐23, IL‐17 and tumour necrosis factor (TNF).

Despite the high efficacy of biologics, their use raises patient and healthcare challenges. These drugs are expensive, so they are used late in the treatment pathway and only in those with severe disease, who represent less than 20% of the total population affected by psoriasis.^3^ Once treatment has started, patients with psoriasis and other immune‐mediated inflammatory diseases remain on costly biologic therapy indefinitely. This contributes to a lifelong healthcare and treatment burden for each individual, leading to concerns due to the uncertainty of the long‐term consequences of sustained immune blockade.

In clinical practice, biologics are prescribed according to ‘one‐size‐fits‐all’ dosing regimens (designed to maximise response at a population level); however, variability in individual treatment response is commonly observed: some patients may require lower or higher doses of biologics to achieve clinical targets. Drug exposure, in part, can explain some of the observed variability in response, and a greater understanding of this relationship may allow for individualised and cost‐effective biologic dosing recommendations. To address this, there is increasing interest in model‐informed precision dosing (MIPD) for biologic therapies in psoriasis.^4^, ^5^, ^6^, ^7^, ^8^ High‐quality pharmacokinetic (PK) and pharmacokinetic/pharmacodynamic (PK/PD) models are essential in supporting these individualised treatment decisions through capture of both drug exposure and clinical effect.

Risankizumab is a humanised IgG1 monoclonal antibody biologic drug that binds to the p19 subunit of IL‐23, thus ameliorating skin inflammation and reducing lesion progression.^9^ It is administered subcutaneously at a fixed dose of 150 mg on weeks 0, 4 and every 12 weeks thereafter.^3^ Risankizumab has a favourable safety profile^10^ and pharmacokinetics typical of biologic drugs: clearance (CL) 0.31 L/day, steady state volume of distribution (V_*ss*_) 11.2 L and half‐life (*t*_1*/*2_) 28 days in a typical individual with psoriasis.^11^ Clinical trials have shown that risankizumab has superior efficacy with respect to resolution of plaques than placebo or other biologics, such as IL‐12/23 inhibitor ustekinumab, TNF inhibitor adalimumab and IL‐17 inhibitor secukinumab.^10^, ^12^, ^13^, ^14^ Trial data indicate that 53‐73% of patients achieve clear or nearly clear skin at week 16 of risankizumab treatment.^13^, ^15^, ^16^

This study aimed to characterise the pharmacokinetics (PK) and pharmacodynamics (PD) of risankizumab in psoriasis using a joint PK/PD model based on real‐world data, capturing a broad population across different stages of the treatment cycle. Drug response was modelled via the Psoriasis Area and Severity Index (PASI). PASI is the gold standard measure of psoriasis severity that encompasses body surface area affected, and grading of redness, thickness and scale of psoriasis plaques at regional sites (head, trunk, arms, legs). PASI is widely used in clinical trials and real‐world practice to assess disease severity and treatment outcomes.^17^, ^18^

We plan to incorporate the PK/PD model into a user‐friendly dashboard to undertake model‐informed precision dosing (MIPD). Our current Precision Dosing Dashboard delivers dosing recommendations based on risankizumab PK^8^; incorporating PD will result in the capture of more heterogeneity in the population for personalised dosing decisions. Understanding the exposure‐response relationship of risankizumab provides an opportunity to facilitate data‐driven care and alleviate the patient and healthcare burdens currently associated with long‐term treatment.

## 2 METHODS

### 2.1 Study design

The dataset was formed as part of the *UK Biomarkers of Systemic Treatment Outcomes in Psoriasis* (BSTOP) study (Research Ethics Committee reference 11/HO802/7), conducted in partnership with the British Association of Dermatologists Biologic Interventions Register (BADBIR). BSTOP is a multi‐centre, prospective, cohort study of real‐world biologic use in psoriasis. Adults with moderate to severe psoriasis requiring systemic therapy were recruited from national specialised services for psoriasis. All participants provided written informed consent prior to enrolment. Adults in the UK who were enrolled in BADBIR and met the BSTOP inclusion criteria^19^, ^20^ were invited to participate. Patients were treated with the standard dosing regimen of risankizumab (150 mg subcutaneously at weeks 0 and 4, followed by every 12 weeks) for chronic plaque psoriasis.

### 2.2 Data collection

Serial serum risankizumab and anti‐drug antibody concentrations were collected during routine clinical visits and analysed using the i‐Tracker assay (Biosynex, Croissy‐Beaubourg, France). Additional serum biomarkers, including albumin and creatinine, were also measured. Clinical assessments included the Psoriasis Area and Severity Index (PASI), along with demographic and clinical data such as age, weight, height, sex and ethnicity. All clinical and demographic data were recorded by attending clinicians.

### 2.3 Pharmacokinetic (PK) analysis

Model fitting was performed using non‐linear mixed effect modelling using nlmixr2 (version 2.1.1.9) in R (version 4.3.1). Model parameters were estimated using the first‐order conditional estimation method with interaction between inter‐individual variability (IIV) and residual variability (FOCE with *η*–*ϵ* interaction). One and two‐compartment models were tested. The objective function value (OFV), a goodness‐of‐fit (GOF) statistic, was used to compare the fit of nested models, where the difference in the OFV (∆ OFV) for models being compared serves as a likelihood ratio test approximately following a chi‐squared distribution. This was supported by GOF plots, consideration of parameter precision and bootstrap analyses. Observations below the limit of quantification (1 mg/L) were handled using Beals M3 method in nlmixr2.^21^

Inter‐individual variability (IIV) for pharmacokinetic parameters was assumed to follow a log‐normal distribution, characterized by exponential random effect models (Equation 1).

(1) $$\theta_{i,k}=\theta_{k}\times {{e^{\eta }}_{i}}^{,k},$$

where $\theta_{i,k}$ is the estimate of the k_*th*_ parameter for the i_*th*_ subject, $\theta_{k}$ is the population estimate of the k_*th*_ parameter and $\eta_{i,k}$ represents the individual deviation from $\theta_{k}$, where $\eta_{i,k}$ is assumed to arise from a normal distribution with a mean of 0 and a variance of $\omega_{k}^{2}:\eta ∼(0,\omega_{k}^{2})$.

Allometric scaling on clearance and volume parameters were collected a priori, with an exponent of 0.75 on clearance and 1 on volume, as well as IIV of clearance. The absorption rate constant (K_*a*_) was fixed from the literature (Suleiman et. al *K*_*a*_ = 0.299)^11^ due to a lack of absorption phase data. Weight was normalized around 70 kg, and where there were enough available covariates, they were normalized around the median (if applicable) and tested for significance within the model (Equation 2).

(2) $$\begin{array}{c} \theta_{i,k}=\theta_{k}\times COV\times cov_{i,z},\\ \theta_{i,k}=\theta_{k}\times (1+COV_{binary}\times cov_{i,binary}), \end{array}$$

where $\theta_{i,k}$ is the estimate of the k_*th*_ parameter for the i_*th*_ subject, $\theta_{k}$ is the population estimate of the k_*th*_ parameter, COV or $COV_{binary}$ represents the covariate parameter, $cov_{i,z}$ represents the individual's normalized covariate (around the median) and $cov_{i,binary}$ is the individual's covariate coded as 0 or 1.

Proportional, additive and combined proportional and additive error models were tested (Equation 3).

(3) $$\begin{array}{rl} C_{i,j} & ={\hat{C}}_{i,j}+\epsilon_{1,i,k},\\ C_{i,j} & ={\hat{C}}_{i,j}\times (1+\epsilon_{2,i,k}),\\ C_{i,j} & ={\hat{C}}_{i,j}+\epsilon_{1,i,k}\times (1+\epsilon_{2,i,k}), \end{array}$$

where $C_{i,j}$ the observed output (serum concentration or PASI) for the i_*th*_ individual and j time, ${\hat{C}}_{i,j}$_*i*_ is the model prediction (serum concentration or PASI) and for the i_*th*_ individual and j time, $\epsilon_{1,i,k}$ is the proportional residual random error for the i_*th*_ individual and j time and $\epsilon_{2,i,k}$ is the additive residual random error for the i_*th*_ individual and j time. Residual random errors were assumed to arise from independent normal distributions with a mean of 0 and a variance of ${\sigma_{n}}^{2}$.

### 2.4 Pharmacodynamic (PD) analysis

PASI was used as a PD marker to assess the disease state. PASI ranges from 0 to 72 and is weighted by area involvement, with higher scores indicating more severe disease. A maximal effect lesion turnover model (E_*max*_) was employed to represent PASI evolution while on treatment, which inhibits the formation of psoriasis skin lesions (K_*in*_ parameter). The EC_50_ parameter represents the half‐maximal effective concentration. Error models, IIV and covariates were tested in the same way as the PK.

The final PK/PD model was utilised to calculate the individual PKPD profiles for 1000 simulated patients based on our real‐world psoriasis cohort (BSTOP) to analyse the exposure‐response relationship. The predicted 28‐week steady‐state serum risankizumab concentration (exposure) and the predicted 28‐week response (reduction in PASI from baseline) were extracted to assess the initial exposure‐response relationship. Four response groups were formed based on this exposure‐response relationship. PASI75 (75% improvement in PASI, compared to baseline) at week‐28 was the high response cut‐off, and the high exposure cut‐off was the 28‐week steady‐state serum risankizumab concentration for 90% probability of achieving PASI90 at month 19 (i.e. 12 months after week‐28). Full responders (at 19 months of treatment) were defined by PASI ≤2 (based on concordance with PASI90^22^), partial responders (at 19‐month) by PASI ≤4 (based on concordance with PASI75^22^) and non‐responders (at 19‐months) by PASI >4. Using the same simulated population and response groups, simulated PKPD parameters (CL, EC_50,_ K_*in*_) were visualized via violin and box plots.

### 2.5 Nomenclature of targets and ligands

Key protein targets and ligands in this article are hyperlinked to corresponding entries in http://www.guidetopharmacology.org, and are permanently archived in the Concise Guide to PHARMACOLOGY 2021/22 (Alexander et al., 2021).

## 3 RESULTS

### 3.1 Study population

Our independent real‐world validation dataset consisted of 50 individuals. Pre‐dose serum drug observations were removed. The final dataset comprised 82 serum risankizumab concentration observations from 48 individuals and 101 PASI observations from 50 individuals (Table 1), where two individuals did not have serum risankizumab concentrations available. All individuals were receiving risankizumab biologic therapy for psoriasis. 16 (20%) serum risankizumab concentrations were below the limit of quantification. All patients had anti‐risankizumab antibody levels below the level of quantification (*<* 10 ng/mL), so this was not tested as a model covariate.

**TABLE 1:** Characteristics of the study population.

Characteristic	Value
Sex (male)	27 (54%)
Ethnicity (White)	34 (68%)
Weight (kg)	79.3 (53, 119)
BMI (kg/m^2^)	26.7 (19.8, 40.2)
PASI at first observation	1.9 (40.8, 0)
Age (years)	47 (19, 75)
Albumin (g/L)	43 (34, 50)
Creatinine (μmol/L)	69.5 (42, 120)
Creatinine clearance (mL/min)	132.4 (6.7, 216.7)

*Note*: Characteristics are presented as median (minimum, maximum) or n (percentage). Total n = 50 unless specified, BMI is body mass index (n = 49). PASI is Psoriasis Area and Severity Index. 3 (6%) patients had concurrent psoriatic arthritis.

### 3.2 Population pharmacokinetic‐pharmacodynamic model

The pharmacokinetic (PK) data were best described by a one‐compartment model with first‐order absorption and elimination with a proportional error model. The addition of a second compartment did not improve model fit. The incorporation of IIV on the volume parameter significantly improved model fit (∆ OFV = −4.6).

**FIGURE 1:**
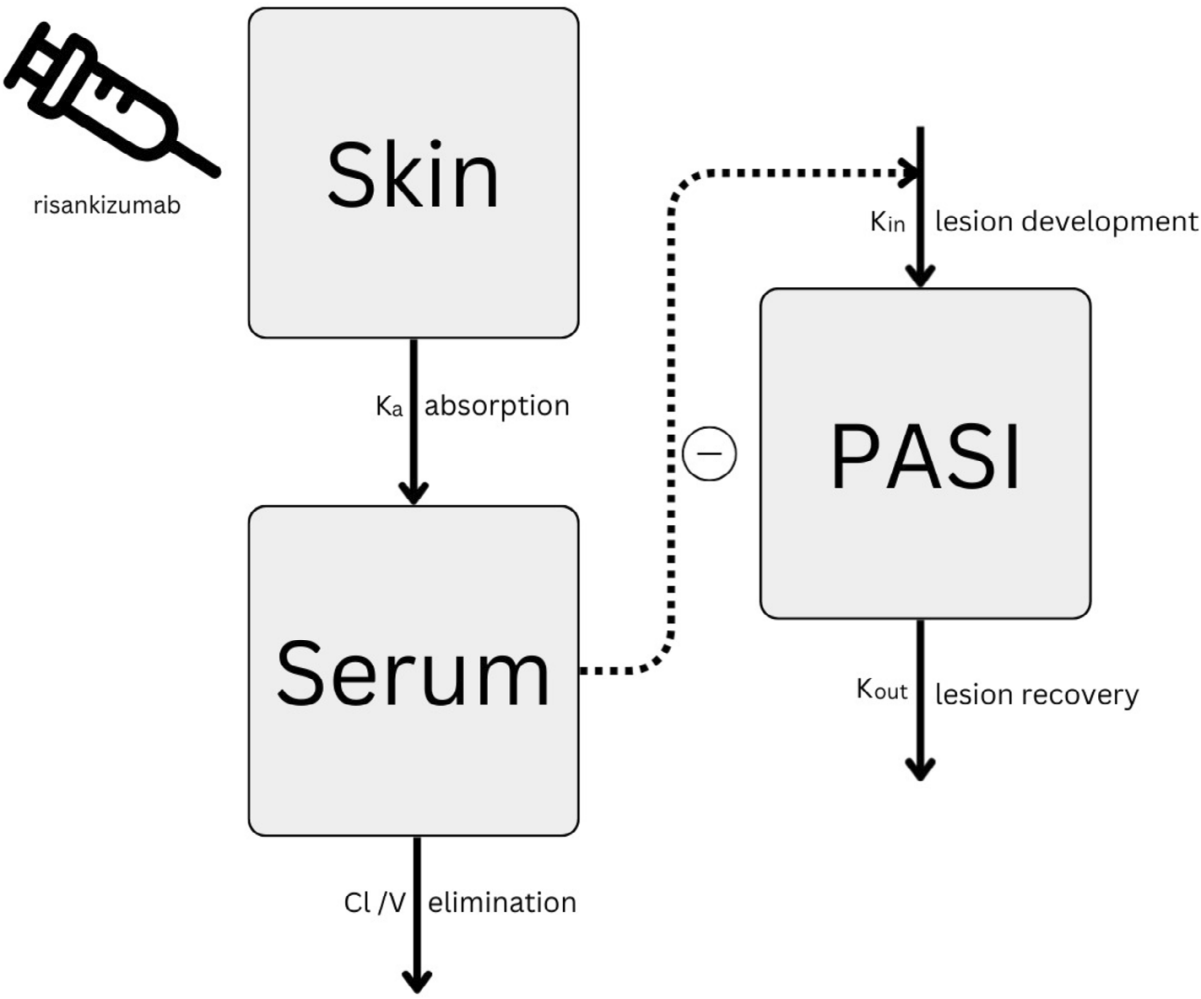
Schematic of risankizumab PK/PD model for psoriasis. K_*a*_ is the absorption rate constant, CL is clearance, V is volume of distribution, E_*max*_ is maximal inhibition, K_*in*_ is lesion development and K_*out*_ is lesion recovery.

Pharmacodynamic (PD) data were described by a turnover mechanism linked with a maximal effect model (Figure 1). GOF plots and relative standard error (RSE) of the final model showed the additive error model best and described the residual variability. The incorporation of IIV on baseline PASI (BSL) significantly improved the model (∆ OFV = ‐97.1), with further improvement following the addition of IIV on EC_50_ (∆ OFV = ‐18.6) and on K_*out*_ (∆ OFV = ‐6.3).

Allometric scaling was collected a priori. No tested covariates were found to be significant (age, sex, albumin and creatinine clearance). Differential equations are presented in Equation 4.

Final model estimates for PK parameters CL/F and V/F were 0.34 mg/L and 12.9 L. PD estimates for BSL, EC_50_ and K_*out*_ were 23.4, 0.11 mg/L and 0.05 day^*−*1^, respectively (Table 2). The final model code is presented in Supplementary Material 1.

(4) $$\begin{array}{rl} \frac{d(\text{skin})}{dt} & =-K_{\alpha }\cdot \text{skin}\\ \frac{d(\text{serum})}{dt} & =K_{\alpha }\cdot \text{skin}-\frac{\mathrm{CL}}{V}\cdot \text{serum}\\ \frac{d(\text{PASI})}{dt} & =K_{\text{in}}\cdot \left(1-\frac{E_{\max}\cdot {\text{concentration}}_{\text{serum}}}{{\mathrm{EC}}_{50}+{\text{concentration}}_{\text{serum}}}\right)-K_{\text{out}}\cdot \text{PASI} \end{array}$$

Final model differential equations, where ${\text{concentration}}_{\text{serum}}=\frac{\text{serum}}{\text{V}}$, Final model differential equations, where ${\text{concentration}}_{\text{serum}}=\frac{\text{serum}}{\text{V}}$, Final model differential equations, where ${\text{concentration}}_{\text{serum}}=\frac{\text{serum}}{\text{V}}$,

**TABLE 2:** Parameter estimates for PK/PD model of risankizumab in psoriasis.

Parameter	Estimate (95% CI)	%RSE	BSV (CV%)	Shrink (SD)%
K_*a*_	0.229 (fix)		
CL*/*F (L/day)	0.341 (0.308, 0.377)	4.77	25.1	39.5
V*/*F (L)	12.9 (11.1, 15.0)	3.05	15.6	73.5
E_*max*_	1.00 (fix)			
BSL (PASI)	23.4 (16.2, 34)	6.01	80.5	27.2
EC_50_ (mg/L)	0.106 (0.0518, 0.218)	16.3	80.5	43.2
K_*out*_ (day^*−*1^)	0.0499 (0.0410, 0.0608)	3.36	30.2	67.3
Coefficients				
coeff_weight on CL_	0.750 (fix)			
coeff_weight on V_	1.00 (fix)			
Error Terms				
prop.err_*pk*_	0.626			
add.err_*pd*_	1.71			

Estimates to 3 significant figures. BSL, baseline Psoriasis Area and Severity Index and model initiation; K_*a*_, absorption rate constant; CL/F, apparent clearance; V/F, apparent volume of distribution; E_*max*_, maximal inhibition; EC_50_, concentration for 50% of maximal inhibition; K_*in*_, lesion development rate; K_*out*_, lesion recovery rate; RSE, relative standard error; BSV, between subject variability; CV, coefficient of variation; SD, standard deviation.

### 3.3 Model evaluation

**FIGURE 2:**
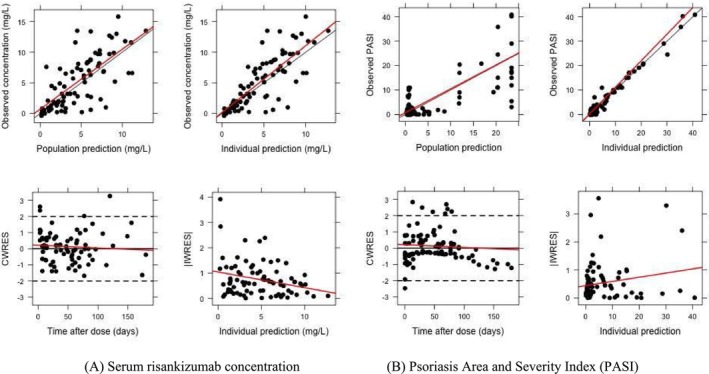
Goodness of fit plots for PK/PD model of risankizumab in psoriasis. CWRES is conditionally weighted residuals and IWRES is individual weighted residuals. Red line is the line of best fit, solid black line is line of identity or y = 0.

**FIGURE 3:**
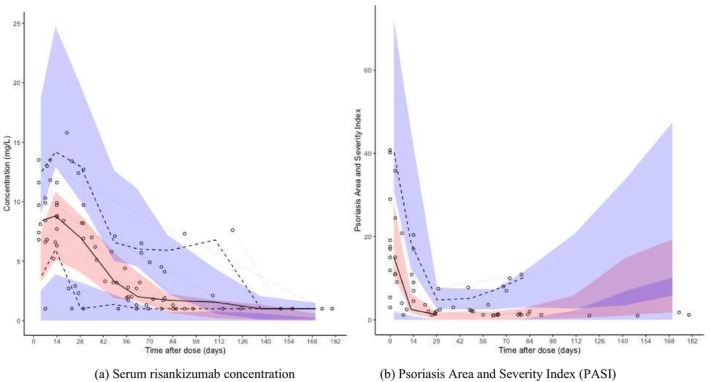
Visual predictive checks for PK/PD model of risankizumab in psoriasis. The black dots are the observed data, and the solid black line is the observed median. The red shading is the 95% confidence interval for the median. The dashed line is the observed 5*th* and 95*th* quantiles. The purple shading is the 95% confidence interval for the 5*th* and 95*th* quantiles.

Goodness‐of‐fit plots showed adequate model fit of the data (Figure 2). No obvious bias or trend was observed with PK or PD data across time or concentration prediction. Visual predictive checks (VPCs) showed a good fit of both serum concentration and PASI, with the majority of data falling within the prediction windows (Figure 3).

### 3.4 Pharmacodynamic analysis

**FIGURE 4:**
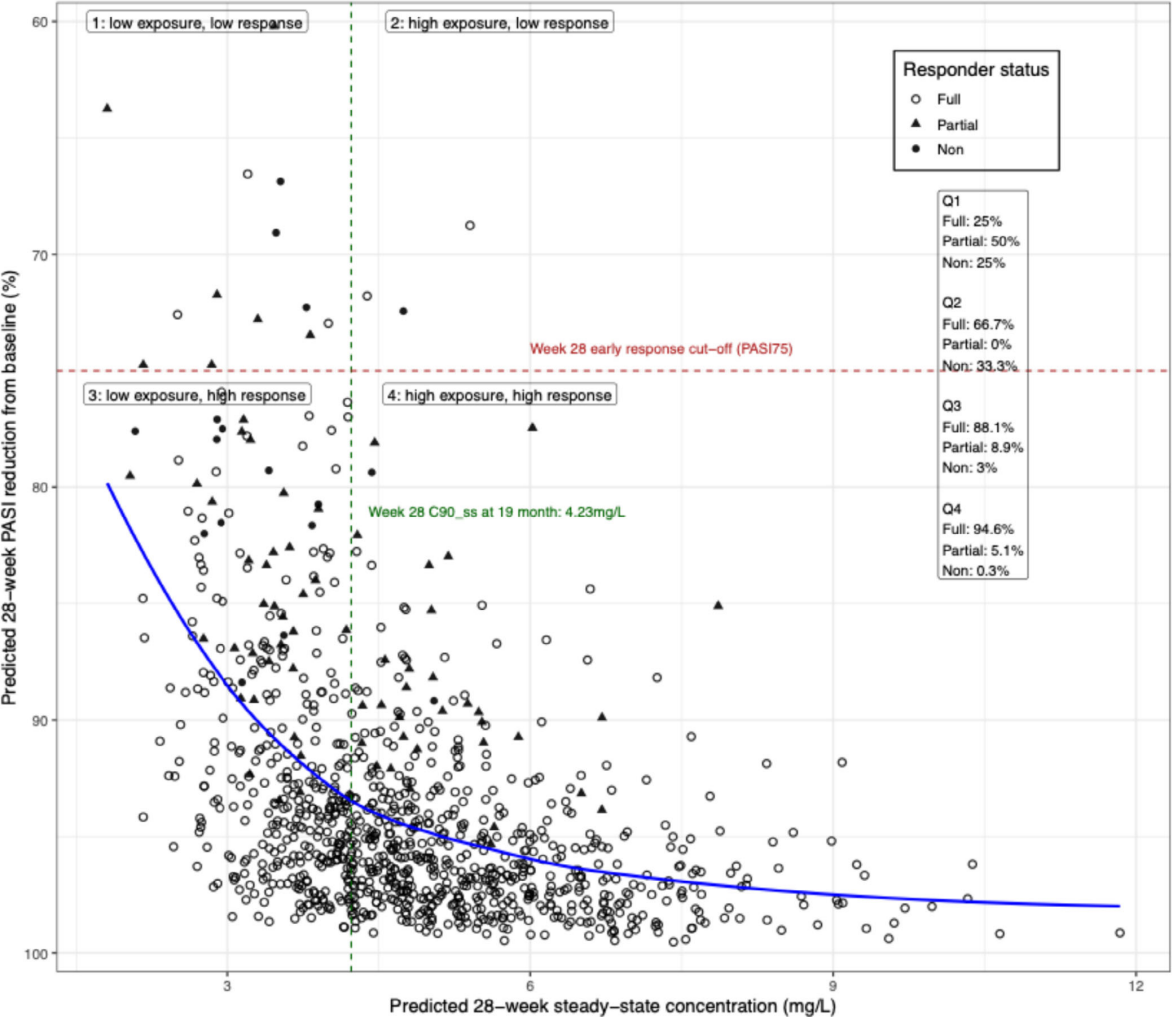
Risankizumab exposure‐response analysis: predicted week‐28 steady state concentration (mg/L) *vs.* predicted week‐28 PASI reduction from baseline/pre‐treatment (%) of 1000 simulated patients with psoriasis using a PK/PD model. Full responders (PASI ≤ 2) at month‐19 are represented by hollow circles, partial responders (PASI ≤ 4 at 19 months) by triangles and non‐responders (PASI > 4 at 19 months) by black circles. Four response groups were formed based on this exposure‐response relationship. PASI75 (75% improvement in PASI during treatment) at week‐28 was the high response cut‐off, and the high exposure cut‐off was the 28‐week steady‐state serum risankizumab concentration for 90% probability of achieving PASI90 at month 19 (i.e. 12 months after week‐28), 4.23 mL/L. quadrant 1 contains simulated individuals with low 28‐week exposure and low 28‐week response. Quadrant 2 contains simulated individuals with high 28‐week exposure and low 28‐week response. Quadrant 3 contains simulated individuals with low 28‐week exposure and high week‐28 response. Quadrant 4 contains simulated individuals with high 28‐week exposure and high 28‐week response. The blue line is the loess line. C90_SS is 28‐week steady state concentration required for 90% probability of PASI90 attainment at month 19.

To gain insight into personalised dosing strategies and explore the potential of model‐informed precision dosing, we performed exposure‐response analysis using 1000 simulated patients with psoriasis based on our real‐world data. Predicted drug exposure and response at week‐28 of risankizumab treatment were extracted. 4.23 mg/L was identified as the week‐28 steady state serum risankizumab concentration for 90% probability of PASI90 response at month 19 (12 months after week‐28) and was used to separate high and low week‐28 exposures. PASI75 was used to differentiate high and low responses at week‐28. At higher predicted week‐28 steady‐state concentrations, greater PASI reduction at week‐28 from baseline was exhibited. The week‐28 high exposure—high response group had the largest proportion of month‐19 full responders (94.6%) and the lowest proportion of month‐19 non‐responders (0.3%), and 5.1% were month‐19 partial responders. The week‐28 low exposure—high response group had 88.1% month‐19 full responders, 8.9% partial responders and 3% non‐responders. The week‐28 low exposure—low response group had 25% month‐19 non‐responders, 50% partial responders and 25% full responders. The high exposure—low response group had 66.7% month‐19 full responders, 0% partial responders and 25% non responders. These groups may provide insight into those who may be suitable for dose de‐escalation or dose escalation. (Figure 4)

**FIGURE 5:**
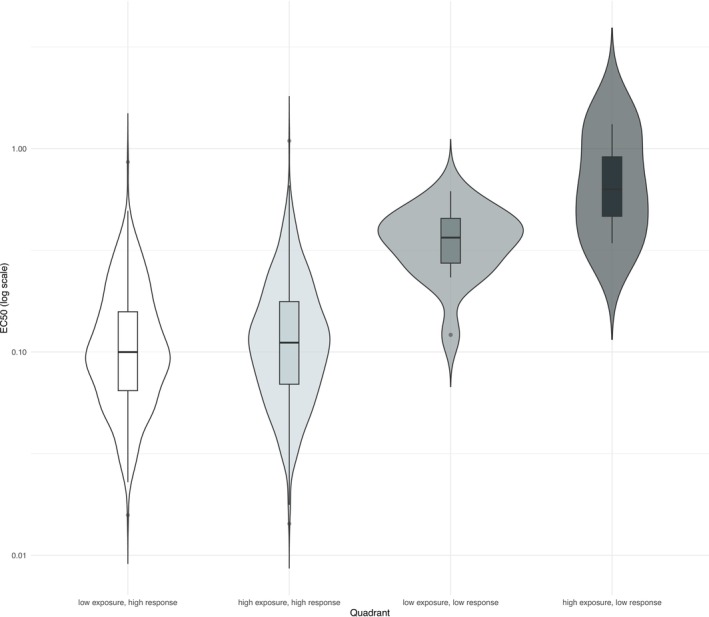
Violin/box plot demonstrating risankizumab EC_50_ values derived from a PK/PD model using 1000 simulated patients with psoriasis. Four response groups were formed based on this exposure‐response relationship. PASI75 (75% improvement in PASI during treatment) at week‐28 was the high response cut‐off, and the high exposure cut‐off was 4.23 mg/L, the 28‐week steady‐state serum risankizumab concentration for 90% probability of achieving PASI90 at month 19 (i.e. 12 months after week‐28). EC_50_, concentration for 50% of maximal inhibition.

Median EC_50_ values for the high response groups were 0.0999 and 0.111 for the low and high exposure groups, respectively. Median EC_50_ values for the low response groups were 0.365 and 0.360 for the low and high exposure groups, respectively. Simulated individuals with low responses require a higher risankizumab concentration for 50% of maximum effect, irrespective of their exposure (Figure 5). K_in_ and CL values aligned closely across groups.

## 4 DISCUSSION

Real‐world pharmacokinetic (PK) and pharmacodynamic (PD) data from 50 patients were successfully evaluated to model the exposure‐response relationship of the exemplar IL‐23p19 inhibitor biologic drug risankizumab in chronic plaque psoriasis. Risankizumab pharmacokinetics were described using a one‐compartment pharmacokinetic model with first‐order absorption and elimination. Psoriasis severity was modelled using the Psoriasis Area and Severity Index (PASI), a gold standard metric, and described via a skin lesion turnover maximal effect (E_*max*_) model. This method captures the complexities of lesion formation across time, providing novel insights into the factors that may be driving variability in treatment response between patients, including drug potency and psoriasis lesion development rate, and potentially serves as a foundation for dosing recommendations for clinical care.

The final model estimates for CL/F and V/F pharmacokinetic parameters of risankizumab were 0.34 L/day, and 12.9 L, where K_*a*_ was fixed (Suleiman et al. K_*a*_ = 0.299). The pharmacodynamic estimates for BSL, EC_50_ and K_*out*_ were 23.4, 0.11 mg/L and 0.05 day^*−*1^, respectively, where E_*max*_ was fixed to 1. Population pharmacokinetic parameters are consistent with previously published risankizumab clinical trial data for moderate to severe psoriasis (CL = 0.24 L/day, Vss = 9.11^11^), active psoriatic arthritis (CL = 0.25 L/day, Vss = 8.97 L^23^) and active Crohn's disease (CL = 0.30, V = 7.68^24^). Between subject variability on CL was similar to those of other risankizumab PK models, and lower for V. In these prior studies, serum albumin and creatinine, CRP and anti‐drug antibody were identified as significant covariates, in addition to sex and age. While we tested these in our real‐world dataset, we did not find statistical significance in our covariate search, likely due to the small and selective nature of our sample. Although comorbidity status was collected, only 3 participants (6%) had concurrent psoriatic arthritis. Due to insufficient data, we were unable to explore this further; however, this remains an interesting area for future research.

Our pharmacodynamic model showcased lesion turnover (*K*_*out*_ = 0.05 day^*−*1^) that was consistent with other turnover models for psoriasis skin lesions developed using datasets of other biologics, such as ustekinumab (0.02 *−* 0.03 day^*−*1^),^25^, ^26^ secukinumab (0.03 day^*−*1^),^7^ adalimumab (0.03 day^*−*1^),^27^ brodalumab (0.05 day^*−*1^),^28^ ixekizumab (0.06 day^*−*1^)^29^ and guselkumab (0.02 day^*−*1^).^30^ This highlights the capture of disease dynamics that can be broadly applied across different drugs. Within our model, where the rate of lesion development (*K*_*out*_) was 0.05 day^*−*1^, a perfectly effective drug (i.e., achieving maximal effect, *E*_*max*_) would result in a lesion half‐life of approximately 14 days. This implies PASI should halve every 2 weeks, corresponding to a PASI50 response, on very effective therapy. After 5 half‐lives (10 weeks), patients should be clear of lesions (PASI100). Across the published models, the estimated lesion turnover rates correspond to half‐lives ranging from 12 to 35 days (*K*_*out*_ 0.06 – 0.02 day^*−*1^).

Between‐subject variability (BSV) in baseline PASI and the concentration required for 50% of the maximal effect was high, at 80.5%. In line with this, high BSV in *EC*_50_ has been reported in literature models for several other biologics, including adalimumab (153%), brodalumab (136%), ustekinumab (287%) and ixekizumab (1660% responders, 581% non‐responders). In the model developed by Pan et al.,^25^ BSV in *EC*_50_ for the single‐population ustekinumab model was 148%. However, this variability was reduced to 43% when a mixture model was applied, identifying two distinct subpopulations. These findings highlight the considerable variability in patient responses, demonstrating heterogeneity in drug potency across the population. Given this variability, PK/PD‐guided dosing strategies like model‐informed precision dosing (MIPD) offer a promising approach to optimising treatment efficacy and identifying potential subpopulations with differential therapeutic responses. This approach has been explored for a wide range of therapies, including antibiotics, oncology drugs, immunosuppressants for transplant surgery and the use of infliximab for inflammatory bowel disease.^4^

Our exposure‐response modelling showed that those with high steady state serum drug concentrations have a greater clinical response, defined by PASI reduction from baseline, and that 28‐week concentrations were predictive of month 19 response. This indicates there may be a role for serum drug concentration‐guided dosing. In those with low exposure and low response, dose escalation may be considered. Equally, individuals with high exposure and high response may be able to dose de‐escalate using MIPD.

Our results also demonstrated that individuals requiring a higher concentration for 50% of the maximal effect (*EC*_50_) tend to have worse responses, indicating PD may also be a useful measure to inform personalized model‐driven dosing. Patients falling into the high exposure—low response group may warrant consideration of switching to an alternative biologic. The low exposure—high response group could be suitable for some degree of dose de‐escalation.

In our exposure‐response modelling, we simulated the 28‐week steady‐state serum risankizumab concentration target required for a 90% probability of PASI90 attainment at month 19. A previous population‐based study by Khatri et al.^31^ modelled the probability of achieving treatment endpoints such as PASI90 using non‐linear regression and population‐level exposure metrics. A population median of individual risankizumab average serum concentration targets of 7.3 mg/L during weeks 0–16 (associated with 77% PASI90 achievement at week 16) and 5.4 mg/L during weeks 16–52 (associated with 85% PASI90 achievement at week 52) were reported. The steady‐state concentration target in our simulated population was 4.23 mg/L. It is important to note that our real‐world study cohort (from which our simulated population was derived) was small and selectively recruited, with greater variability in baseline PASI (PASI at model initiation) (median 1.9; range 0–40.8) compared to Khatri et al.’s clinical trial cohort (mean 20.1; SD 7.7) where patients had moderate to severe psoriasis, which likely contributed to the lower concentration targets observed. This variability supports the utility of MIPD in psoriasis.

The turnover model approach offers a more physiologically representative framework for modelling the dynamics of psoriasis compared to traditional *E*_*max*_ models. By capturing disease progression over time, this method enhances our understanding of longitudinal disease status for informing dosing recommendations. The use of a larger real‐world dataset could further refine model parameters and improve its predictive performance. Additional potential improvements are incorporating Z scores to estimate PASI, as demonstrated by Ooi et al. through the use of a bounded integer model.^32^ While our findings suggest individualised dosing using PK or PK and PD may be an appropriate and exciting avenue, further work on model validation and exploring optimum targets (e.g., area under curve, trough concentration, PASI) is awaited.

Taken together, our PK/PD model for psoriasis adequately described real‐world data. By enhancing our understanding of the relationship between risankizumab exposure and psoriasis severity, we open avenues for personalised biologic dosing strategies. In our previous work, we leveraged PK data using MIPD (and an online dashboard) to inform biologic dosing recommendations.^8^ The current work incorporates PD to further refine these recommendations and capture the variability across the population. This approach enables important diversification across different clinical contexts, including inadequate clinical response as well as complete response (remission), to optimise biologic treatment in a data‐driven way, ultimately promising improved clinical, healthcare and cost outcomes.

## AUTHOR CONTRIBUTIONS

*Conceptualisation*: Satveer K. Mahil, Catherine H. Smith, Joseph F. Standing. *Data curation*: David Baudry, Tejus Dasandi. *Formal analysis*: Charlotte M. Thomas, Jessica Ruoheng Wei; Joseph F. Standing. *Funding acquisition*: Satveer K. Mahil, Catherine H. Smith, Joseph F. Standing, Jonathan Barker. *Investigation*: Monica Arenas‐Hernandez, Jenny Leung, Zehra Arkir, Krystal Rawstron, Chioma Nwaogu, David Baudry. *Methodology*: Charlotte M. Thomas, Joseph F. Standing, Satveer K. Mahil, Catherine H. Smith. *Project administration*: Kingsley Powell. *Resources*: Catherine H. Smith, Satveer K. Mahil, Joseph F. Standing. *Software*: Bola Coker, Charlotte M. Thomas, Joseph F. Standing. *Supervision*: Satveer K. Mahil, Catherine H. Smith, Joseph F. Standing. *Validation*: Charlotte M. Thomas, Joseph F. Standing. *Visualisation*: Charlotte M. Thomas, Joseph F. Standing. Writing—original draft preparation: Charlotte M. Thomas, Satveer K. Mahil, Catherine H. Smith, Joseph F. Standing. *Writing—review and editing*: Charlotte M. Thomas, Satveer K. Mahil, Catherine H. Smith, Joseph F. Standing, Jessica Ruoheng Wei, Jonathan Barker, David Baudry, Tejus Dasandi, Kingsley Powell, Bola Coker, Richard Woolf, Andrew E. Pink, Monica Arenas‐Hernandez, Jenny Leung, Krystal Rawstron, Chioma Nwaogu, Zehra Arkir.

## CONFLICT OF INTEREST STATEMENT

AP has acted as investigator, advisor, speaker or received research or educational funding from Lilly, Pfizer, Abbvie, Sanofi, Almirall, Leo, Galderma, Amgen, Novartis, Janssen, UCB, BMS, BI. CHS reported receiving grants from IMI EU academic industry consortium with multiple partners, the Psoriasis Association, the National Institute for Health Care and Research, AstraZeneca and Boehringer Ingelheim outside the submitted work. The rest of the authors declare no relevant conflicts of interest.

## Supporting information


**Data S1.** Supporting Information.

## Data Availability

The data presented in this study are available at the request of the corresponding author due to ethical and legal restrictions.
