# Supplementary material for: Pharmacokinetic‐pharmacodynamic modelling of risankizumab using chronic plaque psoriasis real‐world data
Source: Br J Clin Pharmacol. 2026 Feb 15;92(7):2136–46. doi: 10.1002/bcp.70477 (PMC13304286; doi:10.1002/bcp.70477)
Supplement: Supplementary file 1 — Data S1. Supporting Information. [file BCP-92-2136-s001.docx]

Supplementary Material

**nlmixr2 model code**

function () {

ini({

######## PK parameters #########

tka <- fix(0.229)

tcl <- log(0.341031)

tvc <- log(12.8803)

######## PD parameters #########

EMAX <- fix(1)

tBSL <- log(23.42055)

tEC50 <- log(0.1062324)

tkout <- log(0.04990573)

######## allometric scalling #########

ALLC <- fix(0.75)

ALLV <- fix(1)

######## inter-individual variability #########

eta.cl ~ 0.0612156340144555

eta.v ~ 0.0239066668906184

eta.bsl ~ 0.499997805486482

eta.kout ~ 0.0870620127168482

eta.ec50 ~ 0.499997805486482

######## error terms #########

prop.err_pk <- c(0, 0.626147813710501)

add.err_pd <- c(0, 1.71375524748229)

})

model({

ka <- (tka)

cl <- exp(tcl + ALLC * lwtz + eta.cl) # lwtz = log(WT/70)

v <- exp(tvc + ALLV * lwtz + eta.v)

BSL <- exp(tBSL + eta.bsl)

kout <- exp(tkout + eta.kout)

EC50 <- exp(tEC50 + eta.ec50)

kin <- BSL * kout

pasi(0) = BSL

d/dt(depot) <- -(ka * depot)

d/dt(centre) <- (ka * depot) - (cl/v * centre)

cp <- centre/v

cp ~ prop(prop.err_pk)

DG <- (EMAX * cp)/(EC50 + cp)

d/dt(pasi) <- kin * (1 - DG) - kout * pasi

pasi ~ add(add.err_pd)

})

}
